# Toward personalized synchrotron microbeam radiation therapy

**DOI:** 10.1038/s41598-020-65729-z

**Published:** 2020-06-01

**Authors:** Elette Engels, Nan Li, Jeremy Davis, Jason Paino, Matthew Cameron, Andrew Dipuglia, Sarah Vogel, Michael Valceski, Abass Khochaiche, Alice O’Keefe, Micah Barnes, Ashley Cullen, Andrew Stevenson, Susanna Guatelli, Anatoly Rosenfeld, Michael Lerch, Stéphanie Corde, Moeava Tehei

**Affiliations:** 1 Centre for Medical Radiation Physics, University of Wollongong, New South Wales, 2522 Australia; 2 Illawarra Health and Medical Research Institute, University of Wollongong, New South Wales, 2522 Australia; 3 Australian Synchrotron - Australia’s Nuclear Science and Technology Organisation (ANSTO), 800 Blackburn Road, Clayton, Victoria 3168 Australia; 4 Central Coast Cancer Centre, Gosford, New South Wales 2250 Australia; 5 Prince of Wales Hospital, Randwick, New South Wales 2031 Australia

**Keywords:** Biophysics, Radiotherapy, CNS cancer, Techniques and instrumentation

## Abstract

Synchrotron facilities produce ultra-high dose rate X-rays that can be used for selective cancer treatment when combined with micron-sized beams. Synchrotron microbeam radiation therapy (MRT) has been shown to inhibit cancer growth in small animals, whilst preserving healthy tissue function. However, the underlying mechanisms that produce successful MRT outcomes are not well understood, either *in vitro* or *in vivo*. This study provides new insights into the relationships between dosimetry, radiation transport simulations, *in vitro* cell response, and pre-clinical brain cancer survival using intracerebral gliosarcoma (9LGS) bearing rats. As part of this ground-breaking research, a new image-guided MRT technique was implemented for accurate tumor targeting combined with a pioneering assessment of tumor dose-coverage; an essential parameter for clinical radiotherapy. Based on the results of our study, we can now (for the first time) present clear and reproducible relationships between the *in vitro* cell response, tumor dose-volume coverage and survival post MRT irradiation of an aggressive and radioresistant brain cancer in a rodent model. Our innovative and interdisciplinary approach is illustrated by the results of the first long-term MRT pre-clinical trial in Australia. Implementing personalized synchrotron MRT for brain cancer treatment will advance this international research effort towards clinical trials.

## Introduction

In the last 30 years, treatment outcomes for brain cancer in children and young adults have remained at a stand-still. Despite significant progress in brain cancer treatment involving surgical resection, radiotherapy and chemotherapeutics, the inherent resistance of these cancers challenge treatment success^1^. The prognosis is even poorer for high-grade gliosarcomas and glioblastoma multiformes (GBMs), and treatments must balance the risk of neurological deficits^2^. Consequently, there has been little improvement in brain and CNS cancer survival between 1990 and 2016 (only −2.2% difference in mortality) despite a 17% increase in incidence^3^. Due to the extremely invasive nature of high-grade brain cancers, treatments remain challenging and research into novel therapies with improved outcomes are still needed.

Synchrotron microbeam radiation therapy (MRT) is an innovative cancer treatment technique proposed in 1992^4^. MRT implements spatially fractionated beams of kilovoltage radiation that are tens of microns in width and spaced hundreds of micrometers apart. The synchrotron radiation source is extremely brilliant and non-divergent, capable of producing a high-flux of photons leading to irradiation dose-rates upwards of thousands of Gray (Gy) per second^5^. The synchrotron microbeam array contains micron-sized beamlets that promote radiosurgical treatment of cancers (with in-beam, or peak doses, of hundreds of Gray). Further, normal tissue sparing is observed, due to the biologically tolerable dose between microbeams (defined as the valley dose). Numerous pre-clinical studies support the reduction in normal tissue damage with MRT, while effectively treating the cancer^5–8^.

Amongst the synchrotron facilities that provide the technical pre-requisites to explore MRT, there is significant variation between treatment techniques including beam dimensions and spacing, beam filtration, image guidance, dose rates and doses. A major uncertainty in prescribing MRT is relating these parameters to systematic tumor control. Early studies^4,9–12^ use skin entrance doses as a standard, providing insufficient knowledge of the tumor dose coverage at depth. A few recent studies^6,12–15^ describe the valley and peak dose in the brain at depth, however, there is scarce individualized tumor volume coverage, as typically used in clinics. Image guidance in MRT is necessary to ensure tumor coverage but is not implemented in all studies. Le Duc *et al*.^16^ is among the few studies to consider co-registration of images and positioning animals accordingly to better target brain tumors. Spatially fractionated MRT doses are challenging to compare with existing modalities. Studies such as Smyth *et al*.^17^ have surmised that the MRT valley dose is the most relatable parameter to standard broad beam treatments, yet the effect of the dose spatial modulation is not well understood.

Furthermore, direct relationships between *in vitro* and *in vivo* MRT studies are scarce. While *in vitro* studies are performed to discern the response of cells to MRT^18,19^, they are not correlated directly to *in vivo* studies. Ideally, as the current focus of clinical practice is personalization, patterns in *in vitro* studies should be used to predict *in vivo* responses in an effort to personalize MRT for better patient specificity. MRT could also benefit from more clinically oriented approaches to treatment planning. The MRT dose coverage of the tumor volume and organs at risk (OAR) must be further investigated. This requires knowledge of the peak and valley dose distribution in the anatomy, and MRT related normal tissue toxicities.

Normal tissue responses to MRT show good tolerance to valley doses greater than 18 Gy^5–7,10,20–24^. However, clinical signs in animals following MRT are not well documented. Brain tumor treatment in human patients can cause adverse effects, including tiredness, skin reactions, headaches, nausea, seizures and hair loss^22^. Previous pre-clinical MRT studies have few reports of early radiation symptoms, and there is no standard for symptom management for brain MRT to-date. No long-term side effects are typically found however, in terms of cell functionality^20^, memory loss^23^, motor function and behavior^24^.

The future of MRT therefore requires the correlation of dosimetry and treatment planning, accurate imaging of brain tumors and image guidance, and reporting of clinical signs and symptom management. To date, there are no pre-clinical studies in MRT that combine the necessary dosimetry, image guidance, treatment planning and short- and long-term follow-up. This study is designed to demonstrate the necessary steps for optimization of personalized pre-clinical MRT of high grade brain cancer: treatment planning, radiobiological insights, image-guidance, and symptom management strategies.

## Methods

### Synchrotron radiation beam configuration and characterization

Irradiations were conducted using the dynamic mode option at hutch 2B of the Imaging and Medical Beamline (IMBL) at the Australian Synchrotron, 34.1 m from the source. The X-ray beam was produced via a 2–3.2 Tesla superconducting multipole wiggler. Full details of beam configurations available at IMBL for synchrotron broad beam (SBB) and microbeams are described by Stevenson *et al*.^25^. Microbeams were produced by passing the beam through a tungsten carbide multi-slit collimator (MSC); 8 mm thick, 40 mm wide and 4 mm high. This produced microbeams (50 µm in width and 400 µm pitch), as described in Stevenson *et al*.^25^. Due to the width of the intrinsic irradiation field size used (10 mm at the sample position), *in vitro* experiments required irradiation of 12.5 cm^2^ flasks in four columns. For *in vivo* experiments, a single column of unidirectional microbeams was used. The complete beam configuration parameters for cell and animal experiments are shown in Table 1, also found in Dipuglia *et al*.^26^.

**Table 1:** Beam configurations for SBB and MRT, *in vitro* and *in vivo* at the Australian Synchrotron IMBL.

Mode	Wiggler Field (T)	Filtration (mm)	Mean energy (keV)	Beam height (mm); Beam width (cm); Number of columns	Intrinsic dose rate (Gy/s) in Solid Water^®^	PVDR	Result Ref.
SBB	***2***	Cu (1.41) Al (2.82)	71.4	0.27;1; 4	40 at 24 mm	N/A	Figs. 4, 5 Table 2
	***3***	Cu (1.41), Cu (1.41)	95	0.49; 1; 4	205 at 24 mm	N/A	Fig. 4 Table 2
MRT	***2***	Cu (1.41) Al (2.82)	71.4	0.27; 1; 4	40 (peak), 5 (valley) at 24 mm	8.4 ± 1	Fig. 5
	***3***	Cu (1.41) Al (2.82)	81	0.5; 0.8; 1	350 (peak), 5 (valley) at 5.5 mm	71 ± 2	Figs. 6–8

All intrinsic dose rates and beam geometry are measured at the sample position. PVDR uncertainty is evaluated within 1 standard deviation. Result references are shown to relate the parameters used to experimental data.

### *In vitro* experiments, dosimetry and treatment verification

The dosimetric protocol for SBB and MRT at the IMBL is outlined in previous publications^25–31^. Briefly, it involves characterizing the pre-filtered SBB uniform in custom designed RMI-457 Gammex Solid Water^®^ phantoms (Gammex-RMI, Middleton, WI, USA) using a PinPoint ionization chamber (IC) (PTW 31014, Freiburg, Germany), calibrated to a traceable standard. A micron-scale spatial resolution X-Tream dosimeter^27,28^ was then calibrated at the same reference conditions: 20 × 20 mm^2^ SBB field at a 20 mm depth. After the insertion of the MSC, the MRT field was characterized using the X-Tream system at 20 mm depth. Microbeam peak and valley doses, and the Peak-to-Valley Dose Ratio (PVDR) were measured for treatment planning. The valley was defined near-midway between microbeam peaks. Final validation was performed with complementary radiation transport simulations before cell and pre-clinical experiments.

Prior to every experiment, the final *in vitro* irradiation doses were verified at the same depth as the monolayer of cells (24 mm) within a 15 × 15 × 15 cm^3^ Gammex solid water^®^ phantom^30^ with a 12.5 cm^2^ cell flask insert that is used to perform the irradiation. For MRT, the X-Tream dosimeter, a PTW microdiamond^31^, and Gafchromic® EBT3 film (24 hour post irradiation analysis only) were used to verify the dose. For SBB, Gafchromic® EBT3 film and PinPoint IC were used to confirm doses. For each irradiation, a film was placed on the back-side (downstream) of the cell flask to confirm the irradiation geometry (SBB or MRT).

### *In vivo* experiments dosimetry and treatment verification

After calibration of the X-Tream dosimeter in the reference conditions, the dose was measured in a 25 × 25 × 50 mm^3^ Solid Water® phantom at 12.5 mm depth for the reference field. The MRT treatment field was further collimated with an 8 × 8 mm^2^ conformal mask and dosimetrically characterized by the X-Tream dosimeter at a 12.5 mm (reference) depth within the phantom. The peak dose, valley dose and PVDR were measured horizontally across 5 central microbeams and associated valley regions at a 5 and 10 μm sampling step size, respectively.

Experimental SBB IC measurements were compared with Geant4 radiation transport simulations modelling the experimental set-up for quality assurance purposes^27^. The Geant4 simulation (version 9.6, patch 4, and using the Livermore Polarized Physics List to model EM interactions) described by Dipuglia *et al*.^26^ was used to evaluate the MRT peak and valley doses at tumor depth. X-Tream measurements were made at a 12.5 mm reference depth in the Solid Water® phantom with precise quantification and tuning of the peak and valley dose using the vertical motor translation speed. Geant4 simulations were then validated with experimental dose measurements at 12.5 mm depth. The dose at tumor depth (5.5 mm) could then be calculated using the Monte Carlo simulation and the vertical translation speed adjusted to deliver the prescribed treatment dose. In this experiment, the prescribed tumor valley dose was 15 Gy.

**Figure 1:**
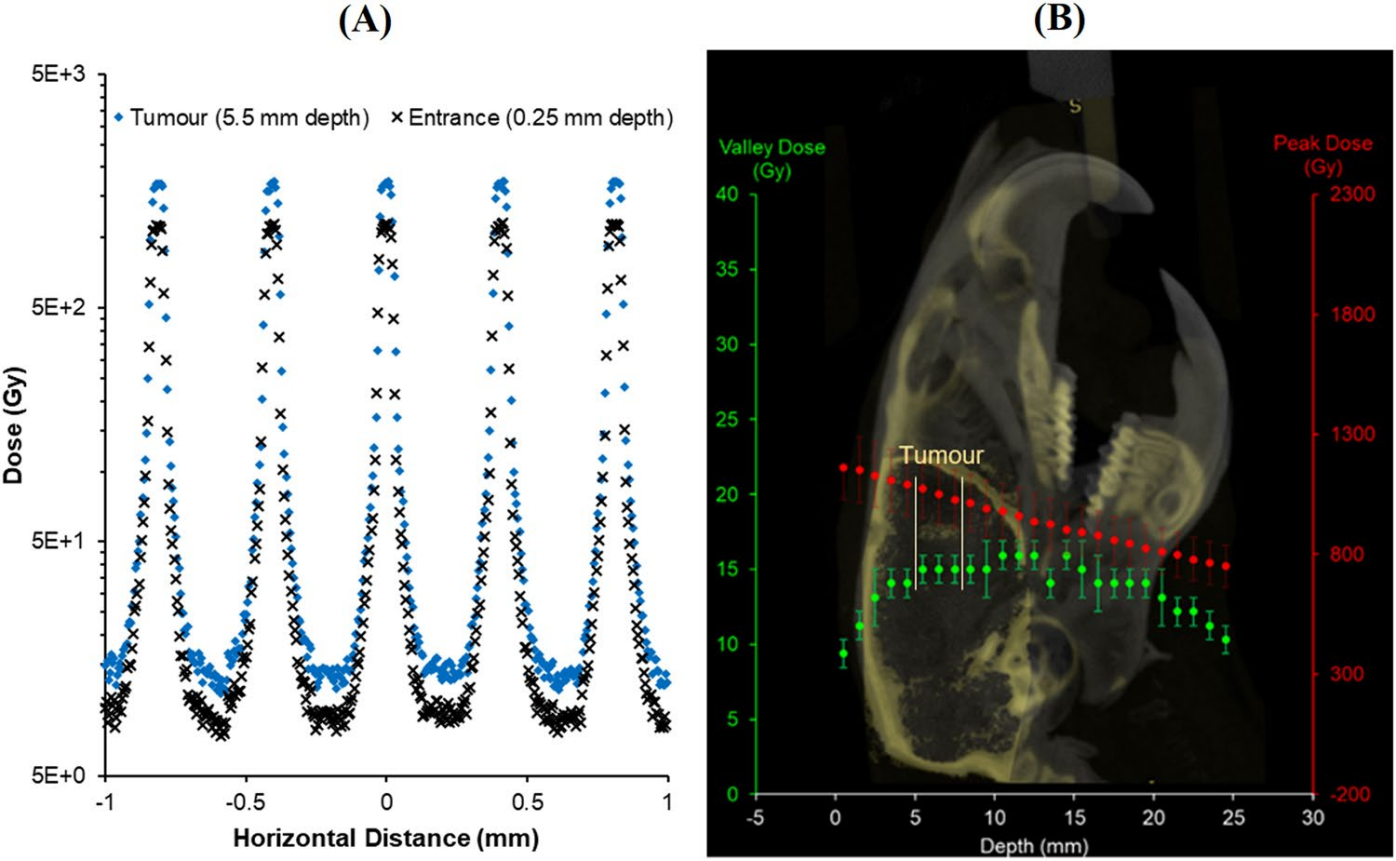
Geant4 Monte Carlo calculated 3 T (Cu/Al) microbeam dose profile (**A**) at the entrance (0.25 mm) and tumor depths (5.5 mm) in water. MRT irradiation depth dose curve (**B**) showing the peak (red) and valley (green) doses in water, overlaid with the micro-CT sagittal profile of a rat in this study to show the dose distribution in the rat and tumor (location indicated by white arrow). Monte Carlo calculated peak and valley doses were verified dosimetrically at 12.5 mm depth.

Figure 1A shows the microbeam lateral dose profile at the entrance and tumor depth, as simulated in Geant4. The peak and valley doses with respect to a range of depths in water are shown in Fig. 1B. This information was used to determine the MRT dose to the tumor and whole brain for a posteriori treatment planning purposes.

### *In vitro* protocols

#### Cell preparation

9 L gliosarcoma (9LGS) is a native gliosarcoma of Fischer rats which contains glial components of glioblastoma multiforme and a sarcomatous component^32^. 9LGS cells were acquired from the European Collection of Cell Cultures (ECACC). The cells were cultured in T75 cm^2^ flasks containing complete Gibco® Dulbecco’s modified eagle medium (c-DMEM), i.e. with 10% foetal bovine serum (FBS) and 1% penicillin and streptomycin (PS). Cells were incubated at 37 ^o^C and 5% (v/v) CO_2_. 9LGS cells were sub-cultured into T12.5 cm^2^ flasks (BD Falcon^TM^) containing c-DMEM prior to irradiation.

#### Cell irradiation

9LGS cells were irradiated at room temperature using clinical orthovoltage or synchrotron X-ray sources. Conventional broad beam (CBB) irradiation of 9LGS was performed at the Prince of Wales Hospital (Randwick, NSW, Australia) using orthovoltage X-rays. T12.5 cm^2^ flasks containing monolayer of 9LGS and 6 mm of c-DMEM were irradiated in horizontal position at a distance of 50 cm from the source in full scatter conditions including Solid Water^®^ below and adjacent to the cells. X-rays were generated using a Nucletron Oldelft Therapax DXT 300 Series 3 Orthovoltage x-ray machine (Nucletron B.V., Veenendaal, The Netherlands). The tube peak voltage was 150 kVp with a beam current of 20 mA, incident on a tungsten target and using downstream filtration of 3 mm Be and additional 0.35 mm of copper and 1.5 mm of aluminium (HVL = 0.68 mm Cu). These X-rays were used to irradiate the cells with a dose rate of 0.76 Gy/min for doses ranging from 1– 8 Gy at 6 mm depth.

At the Australian Synchrotron, cells were irradiated upright in hutch 2B using the dynamic radiotherapy modality at the IMBL for MRT and SBB modes, as given in Table 1. T12.5 cm^2^ flasks containing a monolayer of 9LGS were filled with Hank’s Balanced Salt Solution (HBSS), such that cells were located at 2.4 cm depth. Full scatter conditions were created by using a Gammex Solid Water^®^ phantom material below and adjacent to the flask of cells. Irradiation of flasks was delivered at a minimum scanning rate of 10 mm/s which delivered 1 Gy or 0.4 Gy in the valley to the cells for SBB and MRT, respectively. To cover the 12.5 cm^2^ area of the cells, the treatment was divided into several columns, shown in Table 1. Dose verification was performed as described above.

#### Cell processing following irradiation

For clonogenic assays, cells were washed with DPBS and trypsinized before seeding 3 triplicates at low density into 100 mm petri dishes with 10 mL of c-DMEM. After 15 doubling times, each dish was washed with 5 mL DPBS (with Ca^2+^/Mg^2+^) and stained with a 1:3 (v/v) crystal violet solution with 2.3% crystal violet stock (Sigma Aldrich®) and 70% ethanol. The surviving colonies of 50 cells or more were counted and compared with the initial seeding number to determine the plating efficiency (PE). For each group, the surviving fraction (SF) was calculated by taking the ratio of the PE of the irradiated cells, by the PE of the non-irradiated control. Cell survival data was fitted using GraphPad Prism 7. MRT cell experiments were repeated twice, broad beam 1–4 times. Errors and error bars were evaluated using one standard deviation from the mean.

### *In vivo* protocols

All operative procedures and animal care were in conformity with the guidelines of the Australian Code for the Care and Use of Animals for Scientific Purposes^33^ and under the approval of the University of Wollongong and Australian Synchrotron animal ethics committees agreements (AE17/05 and AS-2017-01).

### Tumor implantation and animal monitoring

A total of ten 7-week old inbred male F344/Arc (Fisher 344) rats from the Animal Resource Centre, Canning Vale, Perth, Australia were housed at the Australian Synchrotron, Clayton, Victoria, Australia in individually ventilated cages containing Pura chips bedding, specialty irradiated feed, access to water and environmental enrichments in groups of 2 or 3. Rats were sub-grouped into MRT-treated or controls, with 5 animals per group.

All rats experienced 1 week of acclimation, before tumor implantation surgery at 8 weeks old. 2 hours prior to surgery, pre-emptive analgesia was provided by voluntary oral administration of 0.4 mg/kg buprenorphine in Nutella (Ferrero Australia Pty Ltd, Lithgow NSW, Australia). Prior to surgery, 9LGS cells were harvested from T75cm^3^ flasks by washing with DPBS, and trypsinizing for 5 minutes. Cells were washed and suspended twice in serum-free DMEM for injection.

Rats were inducted with 5% isoflurane in oxygen and general anesthesia (GA) was maintained with 2.5- 3% isoflurane. Ophthalmic lubricant was applied to protect the eyes and each rat was placed on a heat mat, monitored by PhysioSuite®, (Kent Scientific Corporation, Torrington CT USA). Vital signs including respiratory rate, body temperature and blood oxygen levels were monitored and maintained between 45–65 bpm, 37–38.5 ^o^C, 95–100%, respectively. This ensured all rats recovered from surgery without complications.

Once stable under GA, the scalp was shaved, and the rat was placed on a small animal Kopf Model 900 stereotaxic frame including microinjection unit (Kopf Instruments, Tujunga CA, USA). Bupivacaine was injected subcutaneously in the scalp for local analgesia. A solution of 10% povidone iodine antiseptic was then applied to the scalp and a disposable sterile plastic sheet covered the rat.

An aseptic environment was created to avoid complications post-surgery including sepsis^34^. Surgical drapes, instruments, and equipment and protective gear were autoclaved before surgery, with surgical equipment sterilized for each rat^35^. A dorsal midline incision was made through the plastic sheet commencing posterior to the eyes and extending rostral to the ears. The skull was exposed, and any minor bleeds cauterized. A 0.6–0.8 mm burr hole was made at 3.5 mm to the right of the bregma crossing on the skull using a 1.4 mm K-wire.

The 9LGS cells at a concentration of 10,000 cells per µL were drawn into a 2 µL Neuros Hamilton syringe with a 30-gauge needle (Hamilton Company, Reno NV, USA) and was loaded on the microinjection unit. The syringe needle was inserted through the burr hole to a 6 mm depth into the caudate nucleus of the brain, over 2 minutes. Before injection, the syringe was retracted 0.5 mm to produce a void for the cells. 1 µL of cells was injected with the microinjection unit over 3 minutes. Before withdrawing the needle, cells were allowed to settle for 1 minute. The needle was extracted over 3 minutes. After needle withdrawal, the burr hole was disinfected with alcohol and the wound closed with polypropylene non-absorbable monofilament sutures.

Each rat was given fluid replacement subcutaneously prior to recovery and placed in a warmed recovery cage individually. After 15–20 minutes, fully conscious and mobile rats were returned to their home cage. 12 hours after surgery, another dose of buprenorphine was administered to maintain analgesia. Rats were monitored for post-surgical complications twice daily for 3 days, then once daily for another 11 days.

### Tumor imaging

All rats were prepared for CT imaging using a Siemens Inveon PET/CT Scanner at the Monash Biomedical Imaging (MBI) Facility, Clayton, Victoria, Australia on day 11 post-tumor injection. Prior to imaging, the rats were pre-warmed under a heat lamp before anaesthesia induction using 5% isoflurane. The rats were maintained at 2.5–3% isoflurane for CT imaging, and warmed with a heat mat. Vital signs monitored and maintained.

An iodine contrast agent, Iomeron-350 (Regional Health Care Group, Rosebery, NSW & Bracco Ltd), was used to visualize the tumor against the normal brain tissue. Before iodine injection, the tail was warmed to dilate veins in the tail. The tail was disinfected with water and 70% (v/v) ethanol before a 24 G ¾” Teflon catheter was inserted into the lateral tail vein. 1 mL of iodine was gradually injected (an initial 0.3 mL bolus over 10 s, and the remaining 0.7 mL over 4 minutes) using a pump for imaging during the 8 minute CT acquisition. Rats were positioned on small couch bed with ear bars to keep the skull level in the field. CT was acquired at 80 kVp energy and 200 ms exposure in a 8.7 cm by 8.7 cm field-of-view. Final pixel size was 97 µm. Rats were given oxygen to recover from anaesthesia following imaging, and warmed in isolation, before returning to their home cage.

**Figure 2:**
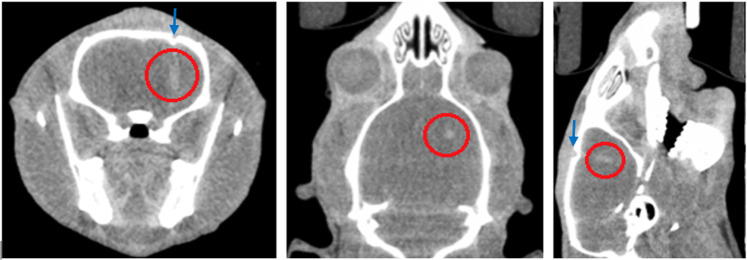
Location of the smallest 9LGS tumor using micro-CT imaging 11 days after injection shown in the red circle in transverse (left), coronal (middle) and sagittal (right) views. Burr hole location is shown (arrow).

The tumor positions were determined with respect to the bony anatomy of the rat. The position of a tumor at day 11 in coronal, horizontal and sagittal views inside the head is shown in Fig. 2 for the rat containing the smallest tumor (surviving rat).

### MRT

On day 12, 5 rats received MRT in hutch 2B on IMBL with each rat receiving 15 Gy in the valley at the target depth of 5.5 mm. 5 rats remain untreated for survival and behavioral comparison. Prior or after MRT, rats willingly ingested 1.5 mg/kg meloxicam as a preventative measure for inflammation following MRT.

**Figure 3:**
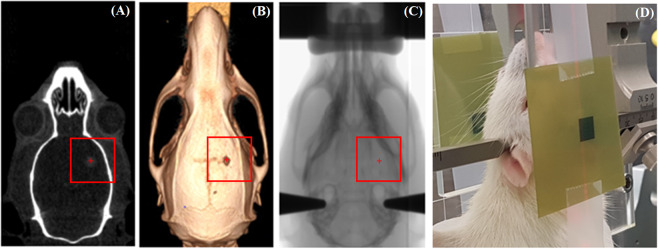
CT anatomical images acquired day 11, and X-ray radiography alignment and MRT irradiation on day 12. Day 11 micro-CT coronal reconstruction showing target outline (**A**), 3D volume rendering of bony anatomy and alignment points (**B**), planar X-ray alignment (**C**), and MRT irradiation with film entry and exit verification (**D**). Red box shows the 8 × 8 mm^2^ MRT delivery field and tumor position (cross) inside (not to scale).

After induction with 5% isoflurane, rats were maintained under general anaesthesia at 2.5–3% isoflurane while warmed with heat mats and heat lamp. Vital signs including respiratory rate and temperature were monitored as previous. Rats were mounted on the Kopf stereotaxic frame without the microinjection unit using a bite block and ear bars. The frame was secured on the treatment stage, securing the rat vertically, with the beam directed through the top of the skull (Fig. 3D). A heat lamp was used for warming during the treatment.

Individual tumors were aligned in the MRT field using an image guidance method developed for pre-clinical radiotherapy applications on IMBL^36^ and the CT image. The IMBL image guidance system (SyncMRT)^36^, was adapted to allow isocentric tumor positioning in the MRT array. 2 planar X-rays of each rat were taken prior to MRT, using an X-ray tube and detected on a Hamamatsu flat panel detector. The total imaging dose of the micro-CT scan and the planar X-ray to the skin was 8.91 ± 0.24 cGy (as determined using a MO*Skin*^TM^ detector^37^).

The X-ray images were then co-registered with the CT image of bony landmarks obtained using the Inveon PET/CT. Landmarks for co-registration between the CT image and the X-ray image were verified within ±0.1 mm, between the anterior and posterior orbits of the eyes, across the bregma line and the length of the skull. Once the X-ray image was aligned with the CT image, the tumor position was targeted by the alignment system. The stage holding the Kopf stereotaxic frame was rotated and translated in 4 degrees of freedom accordingly for each rat to ensure tumor coverage in the 8 × 8 mm^2^ field. This unidirectional field is equivalent to the planning treatment volume (PTV), and fully contains the gross tumor volume (GTV). Figure 3 summarizes the alignment procedure.

Once the image alignment was performed, the rat was treated with MRT. Immediately prior to treatment, 2 pieces of Gafchromic® film were placed anterior and posterior to the tumor position, to verify the MRT irradiation delivery location. The single fraction, unidirectional, MRT delivery was performed using 3 T, 81 keV mean energy X-ray microbeams according to Table 1. Tumor dose coverage was achieved by moving the target (and mask) vertically at a rate of 0.5 mm/s, translating the 0.5 mm high intrinsic MRT field in the cranial to caudal direction on each rat. With this speed, the prescribed dose of 15 Gy in the valley was delivered at the tumor depth, shown in Fig. 1.

As part of the ethically approved animal management plan during the investigation, diazepam was given (4 mg/kg) by intra-peritoneal injection following MRT and after recovery to prevent over-stimulation of rats in recovery.

Rats were monitored for the effects of tumor growth twice daily up to day 200, after which monitoring frequency was changed to once daily. Weight changes, neurological signs, gait, mobility, porphyrin staining, surgical site inflammation, and signs of discomfort were scored. In accordance with humane endpoints defined in the ethics approval, animals were euthanized if scoring exceeded normal values in these categories, or scored cumulatively in neurological signs, gait, mobility surgical site inflammation and weight categories. Death was not used as an endpoint in any part of this project.

### Tissue processing

After euthanasia (following the scoring of clinical signs), the brain of each rat was removed by opening the top of the skull and placed immediately in 10% neutral buffered formalin for immersion fixation. Fixed brains were sliced transversely, processed routinely through graded alcohols and xylene, embedded in paraffin, and 4 µm sections stained with haematoxylin and eosin. Histology images were acquired using 4x magnification on a Nikon® Eclipse TS100 microscope with a Teledyne Lumen*era*® Infinity2 (5 MP USB digital color) microscope camera.

### Retrospective treatment planning with MRT

For the first time, treatment planning has been implemented to describe the long-term survival outcomes following MRT irradiation of 9LGS. Combining all information from the simulation, dosimetry, imaging, and *in vitro* studies, the first patient-specific MRT treatment planning was performed for brain cancer.

CT images of each rat were processed through MATLAB®^38^ to distinguish tumor margins and determine the volume of each tumor as it varies with depth. The 8 × 8 mm^2^ microbeam field entirely covered the tumor in each rat, such that any variation dose coverage occurred only with depth from the surface of the skin. In this way, tumor dose was evaluated for each rat using the predicted Monte Carlo dose, and verified with dose calculations as 12.5 mm depth in Solid Water^®^ only. For this study, cortical bone and realistic rat geometry was not included. Combining this information with the CT derived tumor volume distribution, dose-volume histograms (DVHs) of each tumor were obtained. The whole brain volume (excluding the gross tumor volume) was also considered as an organ at risk (OAR).

## Results

### Cell response to broad synchrotron radiation

**Figure 4:**
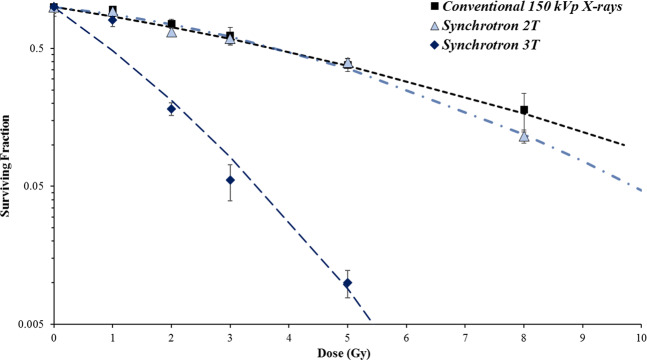
Comparison of synchrotron radiation (with wiggler fields of 2 T and 3 T in Table 1) and conventional orthovoltage radiation on 9LGS cell survival with respect to dose. Errors were determined from the standard deviation of several experiments.

First, the effect of synchrotron radiation on 9LGS cells *in vitro* was compared to conventional orthovoltage X-rays. The major difference between the conventional kilovoltage and synchrotron therapies is the rate at which dose is delivered, as mean energies are comparable (see Table 1). Figure 4 compares the SBB treatments, detailed in Table 1, with the conventional 150 kVp orthovoltage X-rays.

For the same dose range, the impact of increasing the dose rate was synonymous with an increase in 9LGS cell death. The dose rates at the level of the cells using synchrotron 2 T and 3 T wiggler strengths are 40 Gy/s and 205 Gy/s, respectively, compared to the 0.76 Gy/min used with conventional X-rays. Only at a dose rate of 205 Gy/s are significant cell lethality differences noted between the conventional treatment. Cellular dose rate dependence is also seen with FLASH radiotherapy^39^, in favor of normal tissue protection and with equivalent tumor responses. However, our result indicates a greater dose rate influence on the 9LGS cell line, that could produce not only better normal tissue sparing, but radiosensitization of the tumor.

Table 2 summarizes the radiobiological parameters. The most significant change to the radiobiological parameters is the change in the linear component, α. Ranging from 0.124 to 0.740 Gy^−1^, the greatest factor in the change to α is the dose rate of the radiation treatment. The RER_10_ is largest for the high dose rate 3 T treatments due to the dose rate response of the cells and the increase in β also.

**Table 2:** Comparison of all broad beam treatments with regard to radiobiological parameters α and β (according to Eq. 1). The 2 T and 3 T treatment identifiers correspond to the different spectra and dose rate conditions indicated in Table 1.

Treatment	α (Gy^−1^)	β (Gy^−2^)	RER_10_
2T	0.124 ± 0.028	0.0162 ± 0.0042	1.27 ± 0.10
3T	0.740 ± 0.079	0.0371 ± 0.0212	4.07 ± 0.28
Conventional 150 kVp X-rays	0.112 ± 0.030	0.0088 ± 0.0035	1

Dose Rate enhancement ratio (RER_10_) values measured at 10% survival are compared against the conventional 150 kVp X-ray treatment.

### Cell response to synchrotron microbeam radiation therapy

**Figure 5:**
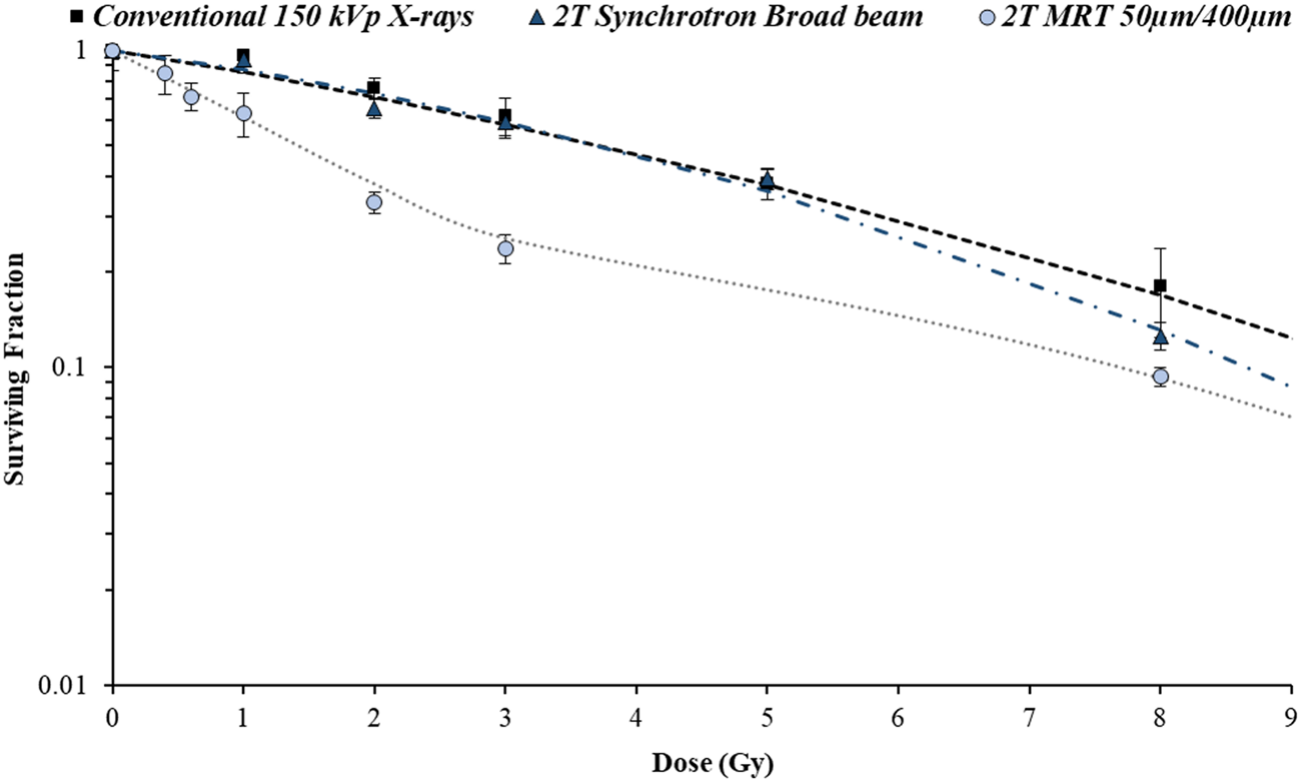
9LGS cell survival using a 2 T wiggler field SBB and MRT (valley dose shown), compared to CBB irradiation. Mean energy of X-rays is 66 keV. Errors were determined from the standard deviation of averages measured over 2 separate experiments. A polynomial fit was applied to the MRT survival curve for visual purpose.

Figure 5 compares the cell survival of 9LGS using CBB, SBB, or MRT using a 2 T wiggler field. MRT produces more cell death than CBB or SBB when evaluating the cell survival against the valley dose. This is due to the increased lethality produced by the peak dose. The change in shape of the cell survival trend does not appear to be linear quadratic when including low doses. The gradient of the survival curve changes and is a function of the individual peak and valley population survivals. At high doses, the cell survival curve tends to follow the trend of the low dose rate SBB or CBB survival of 9LGS, offset by the population of cells that are abolished in the peak.

Overall MRT causes greater reduction in cell survival compared to SBB for the 2 T low dose rate result (Fig. 5). Ideally, for the treatment of 9LGS, a higher dose rate in the valley is required to replicate the important drop in cell survival seen with the 3 T SBB in Fig. 4. Based on these results, the pre-clinical study was optimized in the next section using a 3 T wiggler field, as shown in Table 1.

### Pre-clinical 9LGS Treatment

MRT was performed according to Table 1, with 15 Gy in the valley at the center of the tumor centered in the beam. MRT caused short-term and temporary symptomatic radiation-induced edema in 3 rats, which presented as seizing. Some seizing symptoms were present up 4.5 days after MRT, with the majority of symptoms noticed 2 hours – 1 day after MRT. Meloxicam and diazepam were used for the remaining rats to reduce the risk of seizure activity.

**Figure 6:**
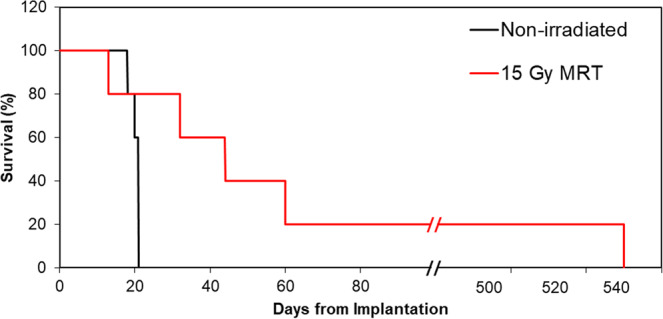
Fischer rat survival post 9LGS implantation for non-irradiated and MRT treated rats with 3 T MRT (see Table 1).

The survival of all rats treated with MRT compared to the non-irradiated group is shown in Fig. 6. MRT treated rats showed significantly longer survival than non-irradiated rats. The mean survival time (MST) and median survival time (MeST) with non-irradiated rats was found to be 20 and 21 days, respectively. In comparison the MST and MeST for rats treated with 3 T Cu/Al, using 15 Gy in the valley, was 135 and 44 days, respectively. The increase in lifespan (ILS) due to the MST and MeST is therefore 570% and 110%, respectively. These results represent the first long-term animal survival study at the Australian Synchrotron. No long-term adverse effects were observed following MRT, and there was no noticeable decline in cognition, vision, mobility, or behavior in treated rats. These observations were made longitudinally twice daily by the same observers. Informal observations were made of rat behavior and ability to learn and remember skills for food rewards (see Supplementary Video). We observed no significant changes to personality or social behavior and were able to remember commands and develop new skills for food before and after MRT. Rat behavior was seen to change only when brain cancer reoccurred.

**Figure 7:**
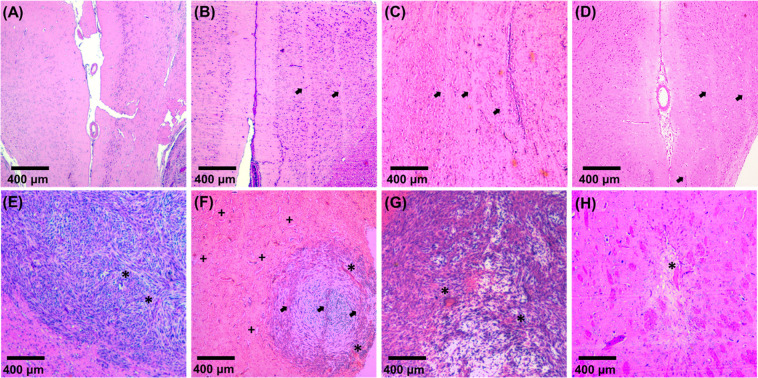
Rat brain histology using H & E staining. The 9LGs tumor was located in the caudate putamen/striatum. The normal tissue was compared in the striatum and cortex. Normal tissue in untreated rat, day 20 (**A**), and MRT treated rats at day 32 (**B**), day 60 (**C**), and day 528 (**D**) after tumor implantation. Untreated tumor at day 20 (**E**), is compared to MRT treated tumors at days 13 (**F**), and 44 (**G**). Complete tumor ablation, leaving only scar tissue, day 528 (**H**). Microbeam tracks indicated with arrows, tumor supporting vasculature (*), tissue vacuolation (+) 1 day after MRT.

Histological analysis of the brain was performed to observe the effect of microbeams on the brain and tumor tissue with respect to time. Figure 7 shows the 9LGS tumor and surrounding normal tissue over time, as stained with H&E.

The histological appearance of the 9LGS tumor in untreated rats was consistent with that in published descriptions^20,39^. Tumors were not encapsulated with locally invasive margins (Fig. 7E–G) and were composed of pleomorphic, occasionally multinucleate spindle cells with a moderate number of mitotic figures. 1 day after MRT (day 13), H&E staining showed dark tumor cells in reoccurring tracks, corresponding to microbeam peak spacing of 400 μm (Fig. 7F). With time and persistent tumor growth, (Fig. 7G), microbeam tracks were no longer present due to repopulation of tumor cells. On closer inspection, cells resembling lymphocytes were detected 1–4 weeks after MRT infiltrating the tumor (Fig. 7G) with some focal edema. In the case of complete recovery and tumor ablation (Fig. 7H), some scar tissue was observed in the region with a large supporting vasculature structure.

In the normal brain, no microbeam tracks were distinguishable using H&E staining 1 day after MRT on day 13 (Fig. 7F), however there was evidence of perivascular vacuolation, indicative of edema. By day 32 (Fig. 7B), microbeam tracks became visible in the normal brain. Between microbeam tracks, normal tissue was indistinguishable from normal untreated brain, highlighting the tissue sparing effect of MRT. Over time (Fig. 7C,D), microbeam tracks become fainter and distorted in normal tissue. At day 60, one rat had small mineralizations (orange in color in Fig. 7C) present throughout the tissue. In the cured rat (day 528), fewer microbeam tracks were seen in the brain. Figure 7D shows some of the more distinguishable microbeam tracks found. Very little mineralization was present and some vacuoles or necrosis were seen where microbeam tracks existed.

Despite deficits in cells along microbeam tracks, no pathologic features were observed in between the microbeams, and the animals did not experience a decline in cognition or signs of abnormal behavior throughout their lives. Cancerous tissue clearance was seen to be aided by heightened immune activity 1–4 weeks after MRT in the form of accumulating lymphocytes within the tumor, which plays a key role in cancer elimination, and has been reported elsewhere^6^. Some features such as collapsed vasculature (Fig. 7C) was noted, and also seen by Barbone *et al*. We did observe some calcification in tissue after 1 year, however, Barbone *et al*. observed significantly more 1.5 months after MRT, perhaps due the larger 9LGS size at the time of treatment (treated day 15).

### Retrospective MRT treatment planning

**Figure 8:**
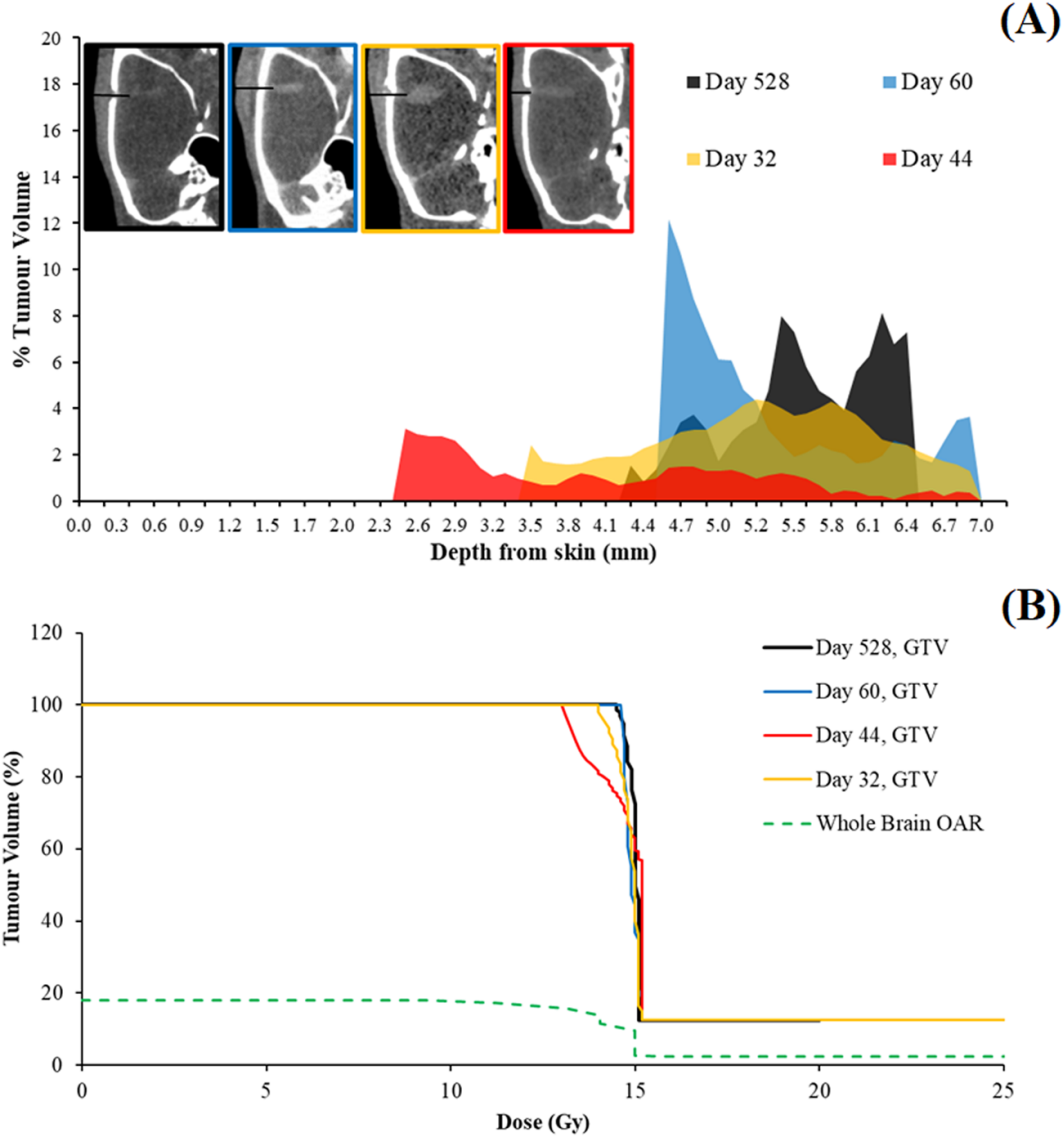
9LGS tumor volume distribution with respect to physical depth from the surface of the skin (**A**) was measured from microCT images (top-left) and used to develop MRT dose volume histograms (**B**) for the gross tumor volumes (GTV) of rats surviving 32 days, 44 days, 60 days, and 528 days compared to the whole brain (OAR). CT datasets were thresholded for the tumor using MATLAB 2018. Limits in accuracy are produced due to the finite pixel size of 97 µm by 97 µm. Monte Carlo evaluated MRT dose with depth was verified dosimetrically at 12.5 mm as described in the Methods.

To understand the factors contributing to successful MRT outcomes, a homogenous treatment planning approach was adopted using the CT data of each rat to determine the tumor volume distribution with respect to depth. This is a first approximation of patient-specific treatment planning for MRT using CT imaging, *in vitro* results, and Monte Carlo dose coverage. The results are shown in Fig. 8 for rats surviving 32, 44, 60, and 528 days after tumor implantation.

Using the volume distribution in Fig. 8A, a dose volume histogram (DVH) was generated for each rat. The dose coverage of individual tumor volumes were computed using the experimentally validated Monte Carlo peak and valley doses. Furthermore, the dose with depth to the brain OAR, was assessed. Figure 8B shows the results for each of the rats in Fig. 8A, including the dose coverage to the brain OAR. Using the tumor DVH, there is a correlation between the tumor-dose coverage and survival in Fig. 8. The best coverage of the tumor volume with 15 Gy resulted in the complete survival case.

Table 3 reviews the tumor coverage shown in the Fig. 8 and evaluates the equivalent uniform dose (EUD). EUD was derived in each case according to Eq. 1, using the simple EUD (Gy) calculation method outlined by Niemierko^40^.

1 $${\rm{EUD}}\,({\rm{Gy}})={{\rm{D}}}_{{\rm{ref}}}\,\mathrm{ln}\left[\frac{1}{{\rm{N}}}\mathop{\sum }\limits_{i=1}^{N}{v}_{i}\cdot {(S{F}_{8Gy})}^{\frac{{D}_{i}}{{D}_{ref}}}\right]/\mathrm{ln}\,S{F}_{8Gy}\,$$

**Table 3:** Summary of rat survival after tumor implantation and other contributing factors including tumor volume, the tumor volume receiving 15 Gy, dose to 90% of the tumor volume and the EUD evaluated using cell survival parameters.

Rat Survival (days)	Tumor Volume (mm^3^)	Tumor Volume Receiving 15 Gy (%)	Tumor Volume Receiving less than 15 Gy (mm^3^)	Dose to 90% Volume (Gy)	EUD (Gy)
**32**	**8.42**	53.58	**3.91**	14.4	15.3
**44**	**1.38**	26.02	**1.02**	13.2	14.6
**60**	**1.72**	44.57	**0.95**	14.7	15.3
**528**	**0.59**	72.22	**0.16**	14.7	15.4

Over each partial tumor volume or voxel, *v*_*i*_, up to a total number of voxels, N, the corresponding dose, *D*_*i*_, is related to a reference dose, *D*_*ref*_, chosen from the *in vitro* MRT data at 8 Gy with corresponding survival (SF_8Gy_). This reference dose was chosen, rather than the previously used 2 Gy reference^40^, to avoid using the *in vitro* data that includes viable cells in the microbeam peak (as described in section *Cell response to synchrotron microbeam radiation therapy*). 8 Gy is also the minimum valley dose tumor voxels may receive.

While tumor volume was related to the treatment success, the overall survival was related to dose coverage. The tumor coverage varied between each rat due to the differences in tumor volumes with depth. As a result, the proportion of each tumor that received 15 Gy varied. This was seen to have consequences for survival when examining the remaining physical volume of 9LGS tumor that received less than 15 Gy. The likelihood for tumor recurrence increased proportionally with the tumor volume not covered by the 15 Gy isodose curve. Other parameters including the dose to 90% of the tumor showed that 14.7 Gy produced the most successful survival outcomes. In reviewing the DVH data, the most important factors in survival appear to be the tumor volume receiving 15 Gy rather than the total tumor volume or the dose to 90% of the tumor volume.

For the OAR, 21% of the whole brain received 8 Gy, with 12% of the whole brain receiving less than 15 Gy. 2.8% of the brain received the peak dose of 800–1000 Gy.

## Discussion

In this study, we developed a new framework for reporting and designing future MRT procedures for brain cancers, which, due to inherent radioresistance, require more rigorous and novel treatment strategies. The *in vitro* irradiation of 9LGS predetermined the tumor response before treating *in vivo*, similar to personalized medicine studies^41,42^. Typically, predicting radio-curability involves analysis of biopsies^43^, or potential biomarkers^42,44–46^. The response of 9LGS towards specific treatments such as MRT or broad beam in our study offered a means of clarifying certain trends in tumor sensitivity to radiation directly before *in vivo* treatment. Conventional kilovoltage radiotherapy of 9LGS (shown previously^47^), identified the significant radioresistance of cells at clinical dose rates (in order of <1 Gy/min). Synchrotron radiation was superior to conventional X-rays in the treatment of 9LGS if delivered at a dose rate of 205 Gy/s. This expands on previous findings^48^, showing that the 9LGS can be radiosensitized using synchrotron high dose rate fields, similar to FLASH therapies^39^.

MRT irradiations of 50 µm microbeams spaced at 400 µm peak-to-peak. Previous studies suggest that MRT survival can be correlated to broad beam conditions using the valley dose^9^, and this approach was adopted for this study. However, the cell survival curves showed two distinct trends of dose-effect relationships, depending on the MRT valley dose. MRT irradiation with a valley dose greater than 3 Gy exhibits a similar trend to SBB; only cells in the valley region are viable, with cells in the peak receiving lethal radiation doses. For valley doses lower than 3 Gy, however, cells within microbeam peaks are viable, further decreasing the overall cell survival compared to SBB for doses <3 Gy. Thus, this dose range is where MRT could be most beneficial compared to broad beam, either as a stand-alone treatment or in combination with other normal tissue sparing strategies. These could include clinical dose fractionation schemes or experimental dose enhancement techniques (such as nanoparticle radiosensitization).

The broad beam dose rate of 205 Gy/s could not be achieved in the valley, due to the beam collimation for MRT. However, this must be considered in the future, to benefit from both FLASH and the radiosurgical properties of MRT. A possible method of implementing this may be through cross-fired or interlaced MRT^49^, but this is at the expense of greater normal tissue exposure. Without dose rate dependence, MRT increased 9LGS cell death compared to lower dose rate broad beam irradiations, SBB (40 Gy/s) and CBB (0.76 Gy/min), due to the radiosurgery of the microbeam peaks.

The *in vivo* MRT results followed the expectations of the *in vitro* experiments and were a product of rigorous dosimetry and tumor alignment with the MRT field.

With a MST of 135 days and MeST of 44 days, we have superior survival to the cross-fired treatment of Le Duc *et al*.^10^ and Regnard *et al*.^17,50^, and some of the unidirectional treatments of Dilmanian *et al*.^16^. However, it is difficult to directly compare between studies due to differing dose rates, beam geometry, tumor volume^51^ and unknown dose coverage of the tumor. The result from Regnard *et al*.^17^ predicts a 12.6 Gy valley dose at tumor depth and has a similar MeST of 40 days to our study, but with less overall survival.

By guaranteeing treatment accuracy and considering patient health, the survival outcomes reflect the expectations of tumor dose coverage of 15 Gy for 9LGS cells and long-term quality of life. The normal tissue sparing and radiosurgery of MRT was verified using histology results (Fig. 7). The microbeams caused an early response in the tumor 24 hrs after MRT, leading to immune-mediated clearance of 9LGS debris 2–4 weeks later. The normal tissue instead recovered over time, to show little difference between untreated and treated brain tissue. Only faint microbeam tracks remain in the brain 72 weeks after MRT. Moreover, MRT made no significant impact on rodent weight gain and growth, or had any noticeable behavioral effects. The 9LGS tumor was cured in 1 rat (which has been rehomed after no cancer was detected 1 year later).

There were, however, differences in the survival that could be a result of disparities in tumor size and location. The previous approach of using a dose of 15 Gy at 5.5 mm depth has been adopted in other studies^6,10,16^, but is not synonymous with treatment success. By considering true dose coverage, using CT imaging and dosimetrically verified Monte Carlo MRT doses, the first individualized treatment planning in MRT for 9LGS was retrospectively performed to understand links between rodent survival and tumor dose-volume coverage. While tumor size was a factor, tumor ablation was ensured if 72% of the tumor volume received 15 Gy, with an EUD of 15.4 Gy. Future studies must include treatment planning before MRT, to assess adequate tumor coverage, and further minimize normal brain (OAR) doses. To remedy the variations in dose with depth, a bolus may be used to increase the depth for shallow tumors. By performing treatment planning and adjustments to tumor depth for each rat prior to treatment, MRT outcomes can be largely improved.

Whilst the survival outcomes were promising in this study, short-term complications occurred, such as temporary symptomatic radiation-induced cerebral edema after MRT. Other treatments have not reported these short-term symptoms, including MRT performed at the European Synchrotron Research Facility (ESRF) using 18 Gy in the valley. What is shown in previous studies such as Fardone *et al*.^24^, is that MRT does not have long term effects on the sensorimotor cortex. The temporary symptoms seen in our investigation therefore may be due to the larger field size or lower dose rate used on the IMBL compared to *in vivo* studies at the ESRF, which may, as a result, produce different normal tissue tolerances. It was further postulated that the use of isoflurane anaesthesia, rather than ketamine-based anaesthesia used in other MRT studies^10,15^, causes more inflammation. Isoflurane provides better recovery but produces higher heart rates and blood pressure compared to ketamine^52^. Isoflurane may also increase the blood oxygen supply to the tumor, hence, the improvement in radiation treatment *in vivo*^53^. The initial inflammation may be reduced if the symptoms can be managed correctly, as no lasting effects in this study were seen with the use of Diazepam and Meloxicam. Anti-inflammatory strategies will be investigated further in future MRT studies at IMBL, to determine management techniques.

## Conclusion

This study provides the first long term MRT animal survival study at the Australian Synchrotron. It identifies the key mechanisms involved in understanding the treatment outcomes of MRT as a brain cancer treatment modality. Thanks to ‘personalized’ *in vitro* cell modelling, pre-clinical MRT was optimized. Using state-of-the-art advanced dosimetry, Monte Carlo, and image guidance, precise *in vitro* and *in vivo* MRT was performed.

Histology results supported the survival data and indicated the stages in tumor control, or repopulation, after MRT, including normal tissue recovery. For the first time, recommendations for analgesia and sedation were reported, to allow for better recovery and symptom management. Individualized treatment planning for brain cancer MRT was implemented to find tumor coverage and other factors contributing to optimum MRT outcomes.

In this study, 8-week-old 9 LGS rats were treated with MRT. Without long-term side effects, this adolescent rat model has promising outcomes for treatment for children with brain cancer, which is often associated with high risks to quality of life. This study highlights the key advantages and strategies for improved brain cancer MRT using a small animal model. Normal tissue sparing, radiosurgical tumor control, and exceptional long-term quality of life were achieved. These developments are the first steps towards personalized clinical strategies using MRT. The extension of this work to larger animals is required, but may ultimately improve the outcome for young patients with brain cancer.

## Supplementary information

Supplementary Video: Rat Behaviour and Skill Development after MRT.
